# Inhibition of MMP-9 by a selective gelatinase inhibitor protects neurovasculature from embolic focal cerebral ischemia

**DOI:** 10.1186/1750-1326-7-21

**Published:** 2012-05-15

**Authors:** Jiankun Cui, Shanyan Chen, Chunyang Zhang, Fanjun Meng, Wei Wu, Rong Hu, Or Hadass, Tareq Lehmidi, Gregory J Blair, Mijoon Lee, Mayland Chang, Shahriar Mobashery, Grace Y Sun, Zezong Gu

**Affiliations:** 1 Department of Pathology and Anatomical Sciences, Center for Translational Neuroscience, University of Missouri School of Medicine, Columbia, Missouri, 65212, USA; 2 Department of Biochemistry, University of Missouri School of Medicine, Columbia, Missouri, 65212, USA; 3 Interdisciplinary Neuroscience Program, University of Missouri, Columbia, Missouri, 65212, USA; 4 MS in Pathology Program, University of Missouri, Columbia, MO, 65212, USA; 5 Department of Chemistry and Biochemistry, University of Notre Dame, Notre Dame, IN, 46556, USA

**Keywords:** Embolic stroke, Focal ischemia, Gelatinase proteolysis, Intracerebral hemorrhage, Neuroprotection, Neurovascular unit, Pericytes

## Abstract

**Background:**

Cerebral ischemia has been shown to induce activation of matrix metalloproteinases (MMPs), particularly MMP-9, which is associated with impairment of the neurovasculature, resulting in blood–brain barrier breakdown, hemorrhage and neurodegeneration. We previously reported that the thiirane inhibitor SB-3CT, which is selective for gelatinases (MMP-2 and −9), could antagonize neuronal apoptosis after transient focal cerebral ischemia.

**Results:**

Here, we used a fibrin-rich clot to occlude the middle cerebral artery (MCA) and assessed the effects of SB-3CT on the neurovasculature. Results show that neurobehavioral deficits and infarct volumes induced by embolic ischemia are comparable to those induced by the filament-occluded transient MCA model. Confocal microscopy indicated embolus-blocked brain microvasculature and neuronal cell death. Post-ischemic SB-3CT treatment attenuated infarct volume, ameliorated neurobehavioral outcomes, and antagonized the increases in levels of proform and activated MMP-9. Embolic ischemia caused degradation of the neurovascular matrix component laminin and tight-junction protein ZO-1, contraction of pericytes, and loss of lectin-positive brain microvessels. Despite the presence of the embolus, SB-3CT mitigated these outcomes and reduced hemorrhagic volumes. Interestingly, SB-3CT treatment for seven days protected against neuronal laminin degradation and protected neurons from ischemic cell death.

**Conclusion:**

These results demonstrate considerable promise for the thiirane class of selective gelatinase inhibitors as potential therapeutic agents in stroke therapy.

## Background

Stroke is a devastating cerebrovascular disease with an estimated 800,000 cases each year and mortality data from 2006 indicated that stroke accounted for approximately 1 out of every 18 deaths in the U.S. [1]. Acute ischemic stroke is the most common form of stroke and is majorly caused by thrombosis or embolism in the cerebral arteries. Blockage of blood flow (ischemia) results in oxygen deprivation, glucose deficiency in the affected region, and infarction in the brain. Cerebral ischemia initiates cascades of pathological events, including vasogenic edema, disruption of the blood–brain barrier (BBB), intracerebral hemorrhage (ICH), astroglial activation, and neuronal death [2,3]. Although the molecular mechanisms underlying these outcomes have not yet been fully addressed, there is considerable evidence supporting the important role of matrix metalloproteinases (MMPs) in mediating ischemia-induced neurovasculature impairment. Increased expression and activation of MMPs, and gelatinases in particular, are likely to play critical roles in excitotoxicity-induced disruption of cell-matrix homeostasis and neuronal cell death [4,5]. MMPs also play a role in thrombolysis-mediated BBB leakage and edema, resulting in ICH. In clinical settings, MMP-mediated neurotoxicity and hemorrhagic transformation during the acute phase of cerebral ischemia are frequently complicated upon treatment with tissue plasminogen activator (tPA) [6]. In addition, gelatinases function in neurovascular remodeling and microvascular recanalization [2,7,8].

MMPs comprise a family of zinc-containing endoproteases that degrade components of the extracellular matrix (ECM) and tight-junctions in the brain. MMPs are also needed for modulating interactions between cells during development and tissue remodeling [5,7]. Reportedly, unregulated MMP activity contributes to neurological disorders, including stroke and other inflammatory responses. MMP-9 activity, in particular, is significantly elevated in humans after stroke [9-11]. High plasma MMP-9 concentrations in the acute phase of a cerebral infarct are considered as independent predictors of hemorrhagic transformation in all stroke subtypes [12]. We and others have shown that aberrant MMP-9 proteolytic activity is associated with an increase in BBB permeability, which results in brain edema and hemorrhage, and contributes directly to neuronal injury, apoptosis and brain damage after acute cerebral ischemia [13-16]. Moreover, cerebral infarct size is reduced in mice deficient in MMP-9, or after treatment with MMP inhibitors [17].

Among the different cell types in the neurovasculature, pericytes are known to play a role in safeguarding the brain against injury, and control key neurovascular functions and neuronal phenotype in the adult and aging brain [18-20]. Loss of pericytes, associated with accumulation of neurotoxic and vasculotoxic macromolecules in the brain, could reduce brain microcirculation, diminish brain capillary perfusion, and disrupt the BBB, leading to vascular damage. Analysis of pericyte-deficient mice (e.g., with *Pdgfb* mutants) reveals that pericyte deficiency leads to increased permeability of the BBB, which probably occurs by facilitating endothelial transcytosis [18]. Although MMP-9 inhibition or knockout can attenuate proteolysis of BBB [13,17], more recent studies suggest its possible role in neurovascular regeneration, especially in the delayed phase of cerebral ischemia [21]. Thus, successful anti-stroke therapies require selective inhibition of aberrant activity without altering the physiological function of MMPs, such as their roles in axonal growth, synaptic plasticity, and vascularization in the central nervous system [22-26].

Pathologically activated therapy (PAT) is a novel neuroprotective strategy based on the principle that drugs are activated during the pathological state of the target, while sparing normal tissue function [27]. One PAT strategy is the target-induced activation of MMP inhibitors. Specifically, (4-phenoxyphenylsulfonyl)methylthiirane (referred to as SB-3CT) is the first mechanism-based MMP inhibitor selective for gelatinases [28]. Active gelatinases bind to SB-3CT and catalyze the opening of the thiirane ring in the molecule. The resultant species generated within the active site of the enzyme affords tight binding between the inhibitor and the enzyme. That is to say, the activity of the enzyme generates the potent inhibitory species within the active site. This mechanism-based inhibition confers a PAT strategy to abrogate the deleterious activity of gelatinases, making this class of inhibitors potentially suitable for more prolonged treatment, because of the selectivity that it affords. We first reported that SB-3CT prevents proteolysis of the ECM basement membrane laminin and rescues neurons from focal cerebral ischemia [29]. Most importantly, significant therapeutic benefit can be observed for up to 6 hours after initial injury. Recent studies show that SB-3CT abolishes oxygen-glucose deprivation-induced reduction of the tight junction protein occludin as well as decreases Evans blue extravasation and apoptotic cell death after spinal cord injury, subarachnoid hemorrhage [30-32], and cardiopulmonary resuscitation-induced BBB disruption [33]. These results are consistent with the notion that inhibiting pathological gelatinolytic activity can maintain the brain neurovascular integrity and protect it from ischemia or other neurovascular insults.

In the present study, we use a fibrin-rich blood clot to induce middle cerebral artery (MCA) occlusion in mice. This embolus-induced focal cerebral ischemia model is more physiologically relevant to thromboembolic stroke in humans. We demonstrate the protective effects of SB-3CT on the neurovascular unit, and document that SB-3CT can manifest inhibitory activity even in the presence of an embolus blocking the MCA and its branches. We disclose that repeated-dose administration of SB-3CT over seven days in the embolus-induced “permanent” focal cerebral ischemia model also protects the brain from neurovascular impairment with no apparent cytotoxicity.

## Results

### Embolus-induced focal cerebral ischemia in mice

**Figure 1:**
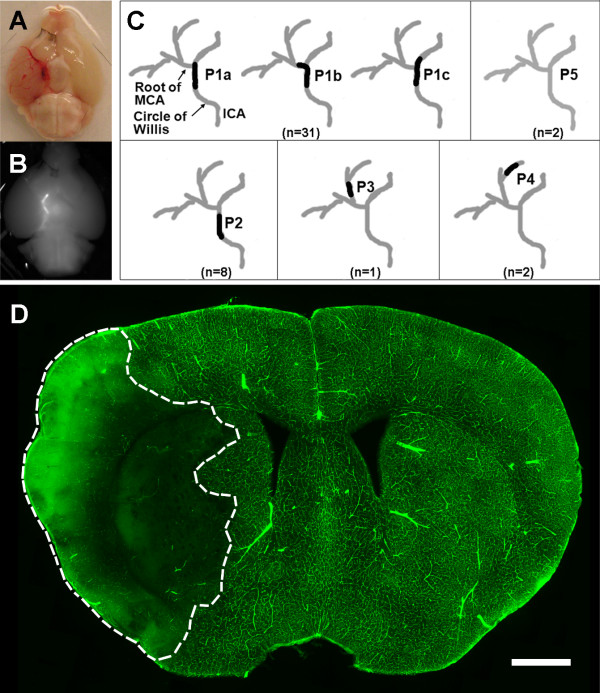
**Patterns of embolus blocking the MCA. a** A representative picture showing the site of the embolus blocking the right MCA at the circle of Willis. After injection of clot, mice were transcardially perfused with saline and the brain was removed. **b** The position of embolus was confirmed by injection of clot labeled with fluorescent dye, marking blockage of the right MCA. **c** Identification of embolus positions (P) in brain 24 hours after ischemia. P1 (P1a–P1c together) indicates complete blockage of the MCA, P2 indicates that an embolus did not reach the MCA, P3 indicates that an embolus was lodged in the second branch of the MCA, P4 indicates that an embolus was lodged in the third branch of the MCA, and P5 indicates that no embolus was seen. Number of animals (n) in each position is shown in parenthesis. **d** Validation of embolus-induced MCA blockage by FITC-dextran infusion. A representative coronal section from a brain after venous infusion of FITC-dextran showing infarct area in the right MCA supplied regions (marked by dashed white lines) 24 hours after embolus-induced ischemia. Perfusion of FITC-dextran marks the microvessels in the unlesioned brain regions. In the peripheral part of the infarct cortex, high intensity of FITC-dextran indicates leakage of the 2,000 kDa macromolecule from microvessels into the infarct area. Section thickness, 100 μm; Scale bar, 800 μm.

To evaluate the effects of the selective gelatinase inhibitor SB-3CT, we employed a modified version of a previously described embolic focal ischemic model [34]. This model was created by delivery of a 10-mm long, fibrin-rich autologous clot into the stem of the right MCA. To ensure MCA occlusion and to determine distribution of the embolus lodgments, we harvested the brains with intact vasculature 24 hours after clot injection and examined the patterns of the embolus blocking the right MCA (Figure 1A). Injection of a clot labeled with fluorescent dye marked the location of the embolus within the circle of Willis at the origin of the right MCA (Figure 1B). In most cases (31 out of 44; >70%), the embolus was found to occlude the right intracranial segment of the internal carotid artery at the origin of the MCA, designated as position P1 (Figure 1C). In a few cases (3 of 44; <7%), the embolus occluded the MCA at further branches at positions P3 to P4. The occlusion at position P2 (8 out of 44, 18.2%) indicated that the embolus did not reach the origin of the MCA. In rare cases (2 of 44; 4.5%), clots were not found (P5 in Figure 1C). This is likely due to inherent variations in the procedure of embolus preparation. We have eliminated variations by standardizing the washing steps and selecting homogenous, smooth and well-formed clots for MCA injection. 

Next, we examined the embolic blockage of the MCA-supplied microvasculature by intravenous infusion of a fluorescence-labeled macromolecule. Confocal microscopy with fluorescein isothiocyanate (FITC)-dextran (2,000 kDa) revealed the embolus-blocked microvasculature as marked by dashed white lines in a representative serial brain section, indicating the presence of infarct (Figure 1D). As expected, the contralateral side (left intracranial segment) of the internal carotid artery was unaffected. Examination of the embolic region by cresyl violet staining revealed extensive cell death with small-condensed nuclei in the ischemic hemisphere (data not shown).

**Figure 2:**
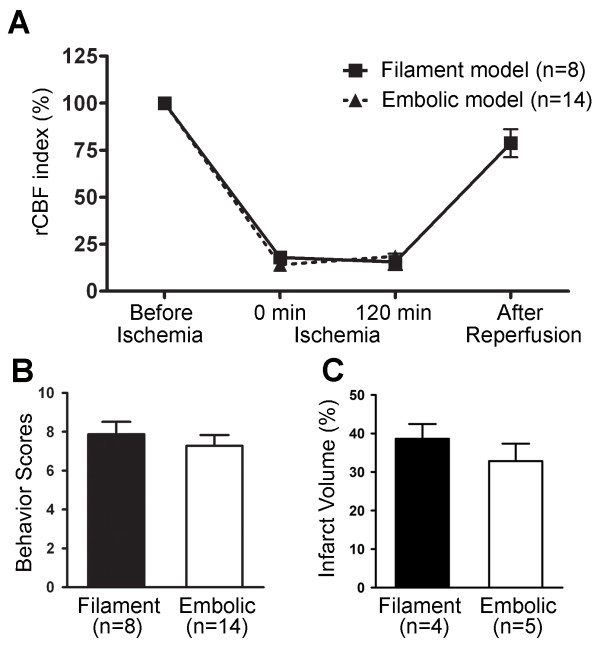
**Comparison of the embolic versus filament ischemia models. a** rCBF index before and during ischemia, as well as after reperfusion. There was no significant difference in rCBF in two-hour ischemia between mice with either type of ischemia. rCBF showed a 75% reduction relative to pre-ischemic baselines in 79% (68 out of 86) of the animals with embolic ischemia, comparable to the previous reports [29,34-36]. **b** Behavioral tests of motor, sensory and reflex function scored using the mNSS on a scale from 0 to 14 (0 = normal, 14 = maximal deficit). Analysis indicated no statistically significant difference between embolic and filament models; *p* > 0.05, one-tailed Student’s *t*-test for the sum of behavioral scores. **c** Infarct volume quantified by 2,3,5-triphenyl-tetrazolium chloride (TTC) staining. No statistically significant difference was seen in infarct volumes in the two models; *p* > 0.05, by one-tailed Student’s *t*-test. Number of animals (n) in each group is shown in parenthesis, and data are expressed as means ± SEM. These results indicate that both focal cerebral ischemia models are comparable.

Moreover, we compared the mouse embolus-induced MCA occlusion to the filament model in which a filament was inserted for two hours followed by reperfusion. Reports from our laboratory and others have indicated that transient MCA occlusion longer than two hours leads to considerably large infarct volumes in the cortex, as well as neuronal death in the hippocampus and striatum of the affected hemisphere [2-4]. This model also shows impairment of neurovasculature, as well as an identifiable penumbra of astrogliosis (data not shown). In both the embolic and filament models, there were similar declines in regional cerebral blood flow (rCBF) levels to 25% of baseline and no significant difference in rCBF was observed during the two-hour ischemia phase (Figure 2A). Similarly, comparable neurobehavioral scores and infarct volumes were observed 24 hours after both embolic and filament-induced MCA occlusion (Figures 2B and 2C). 

### SB-3CT reduces infarct volume and ameliorates behavioral functions after embolic cerebral ischemia

**Figure 3:**
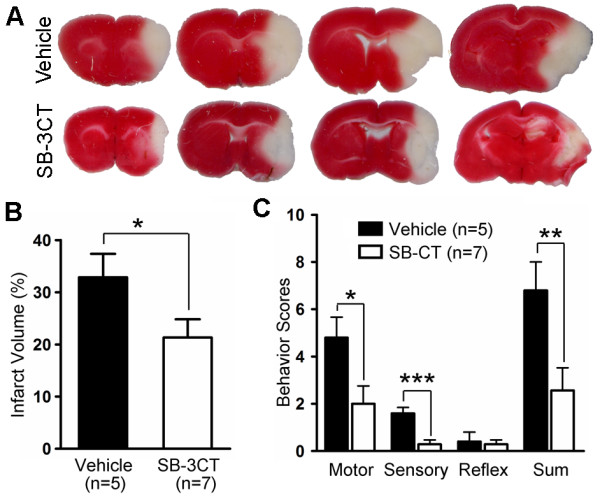
**SB-3CT protects against brain damage and ameliorates neurobehavioral deficits after embolic MCA occlusion in mice.** Mice were injected ip with SB-3CT (25 mg/kg body weight) or vehicle 2 and 4 hours after embolus-induced focal cerebral ischemia. Neurobehavioral tests were conducted 24 hours later and scored using the 14-point mNSS, followed by assessment of infarct volume by TTC staining. **a** Representative images of TTC staining. **b** Quantification of infarct volumes analyzed by TTC staining using ImageJ tools. *, *p* < 0.05 by one-tailed Student’s *t*-test. **c** Improvement in motor and sensory function following post-ischemic SB-3CT treatment compared to the vehicle-treated controls; *, *p* < 0.05 for motor; ***, *p* < 0.001 for sensory; and **, *p* < 0.01 for the sum of the behavioral scores, respectively, by one-tailed Student’s *t*-test. Number of animals (n) in each group is shown in parenthesis, and data are expressed as means ± SEM, whereas reflex activity was unchanged.

We previously reported that SB-3CT could rescue neurons from transient focal cerebral ischemia [29]. Here, we evaluated the efficacy of SB-3CT in the embolic model after intraperitoneal (ip) administration at 25 mg/kg at 2 and 4 hours post-ischemia, and examined neurobehavioral outcomes and potential protective effects against brain damage. As previously observed, SB-3CT treatment did not alter physiological indices, including arterial blood gas and glucose levels, as compared to vehicle-treated animals [29]. The mean infarct volume in vehicle-treated mice, as indicated by staining of brain sections with 2,3,5-triphenyl-tetrazolium chloride (TTC, Figure 3A), was 33% of the total hemisphere after accounting for edema (Figure 3B) [17,34,37]. Post-ischemic treatment with SB-3CT significantly diminished brain damage and reduced infarct volume to about 20% of the total hemisphere (Figure 3B). Neurobehavioral outcomes based on motor, sensory functions, and reflex activity were assessed at 24hours using the 14-point modified neurological severity scoring (mNSS) measurement protocol (see Methods) [37]. SB-3CT treatment ameliorated neurobehavioral outcomes, particularly in the motor and sensory functions (Figure 3C). These findings indicate that SB-3CT confers protection to brain cells, despite the presence of the embolus. 

### SB-3CT inhibits MMP-9 activity in the embolus-induced ischemic brain

**Figure 4:**
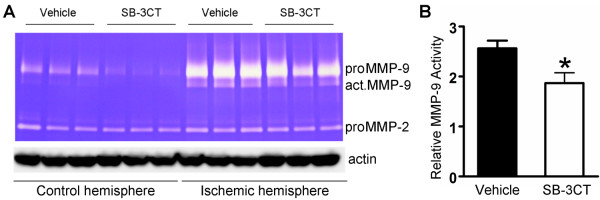
**SB-3CT inhibits elevated MMP-9 activity after embolus-induced MCA occlusion in mice. a** Gelatin zymography reveals increased levels of MMP-9 zymogen (proMMP-9) and activated (act.MMP-9) enzyme in embolus-induced focal cerebral ischemia, and SB-3CT treatment attenuated the increase in proMMP-9 and act.MMP-9. Under these experimental conditions, proMMP-2 was not altered. **b** Quantification of relative MMP-9 gelatinolytic activity (n = 3) *, *p* < 0.05 by one-tailed Student’s *t*-test; data are expressed as means ± SEM.

To determine whether SB-3CT administration inhibits gelatinolytic activity after embolus-induced focal ischemia, we examined the brain tissues by gelatin zymography. The analysis revealed increases in proMMP-9 and active MMP-9 levels in the ischemic hemisphere after embolus-induced MCA occlusion. SB-3CT treatment attenuated both proMMP-9 expression and the level of active MMP-9 (Figure 4). Although the mechanism for attenuation of proMMP-9 expression by SB-3CT is not well understood, there is evidence of a feedback loop for modulation of the proMMP-9 expression by transcription factors such as NF-kappa B and AP-1 [5]. Under these conditions, we did not observe any variation in the levels of the proform of MMP-2 (proMMP-2; Figure 4A). This is consistent with previous findings of the presence of constitutively expressed proMMP-2 and inductively expressed MMP-9 in transient ischemia or spinal cord injury models in rodents [29,30], and in humans after the onset of stroke [9-11]. 

### SB-3CT protects laminin-positive pericytes from embolus-induced ischemic lesion by relieving them from gelatinase-mediated lumen contraction after embolic MCA occlusion

**Figure 5:**
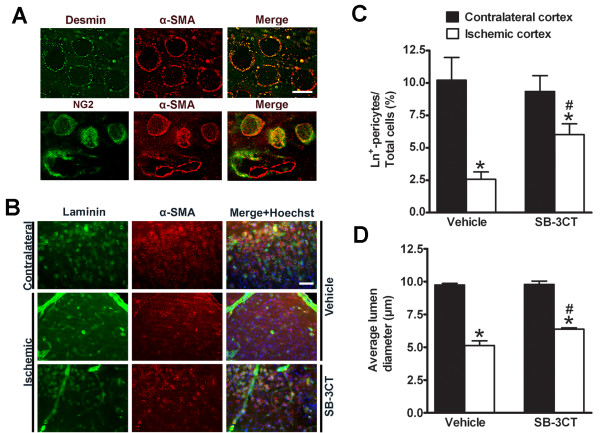
**SB-3CT protects pericytes from ischemia-induced contraction and cell loss after embolic MCA occlusion in mice. a** Colocalization of α-SMA-positive immunoreactivity with pericyte markers NG2 or desmin in the unlesioned cortex. NG2 or desmin monoclonal antibody (mAb) was used to detect pericytes with anti-mouse IgG-Alexa488 (green), followed by immunostaining with α-SMA mAb which was visualized by anti-mouse IgG-Alexa594 (red). Scale bar, 8 μm. **b** Representative images of ischemic cortex penumbras as compared to the corresponding contralateral regions in mice treated with SB-3CT or vehicle-treated control. Vascular cells showing colocalized (yellow) staining are laminin-positive pericytes. Top row: Pericytes identified by α-SMA appear as annular cells surrounding the lumen in the non-ischemic cortex. Middle row: Constricted vascular lumens were surrounded by contracted α-SMA immunostaining. The number of laminin-positive pericytes in the ischemic region was significantly decreased compared to the non-ischemic cortex. Bottom row: Contraction of pericytes and loss of laminin-positive pericytes were protected by SB-3CT treatment. Scale bars: 50 μm. **c** Quantification of the percentage of laminin-positive pericytes in cortex after treatment with SB-3CT or vehicle. Data were analyzed by one-tailed Student’s *t*-test: *, *p* < 0.05 compared ischemic hemisphere to contralateral hemisphere (paired *t*-test); #, *p* < 0.05 compared the difference between contralateral and ischemic hemisphere after SB-3CT treatment to that of the vehicle-treated control (unpaired *t*-test). **d** Quantification of the mean diameters of pericyte-encircled lumens. The average lumen diameter was calculated in approximately 300 pericytes from 4–5 randomized cortical areas of the ischemia penumbral region and the corresponding contralateral hemisphere in each brain (n = 3 in each group). Pericyte-encircled lumen diameters in the ischemic cortex were compared to those in the contralateral side treated with SB-3CT or vehicle. *, *p* < 0.001 compared to the non-ischemic cortex; #, *p* < 0.05 compared the difference between contralateral and ischemic hemisphere after SB-3CT treatment to that of the vehicle-treated control; data in panels C and D are expressed as means ± SEM.

We have previously shown that degradation of laminin after focal cerebral ischemia involves MMP-9 activation [29]. Other studies have also shown that pericytes play a role in constriction of the vascular wall, leading to obstruction of capillary blood flow during brain aging or ischemia in the adult brain [19,20]. To investigate the effects of SB-3CT on gelatinase-mediated proteolysis of laminin after embolic ischemia in mice, we used triple-color confocal imaging of the well-established pericyte markers α-smooth muscle actin (α-SMA), chondroitin sulfate proteoglycan NG2, and desmin. We also immunostained laminin, an ECM component, to visualize brain capillary vascular profiles and then examined brain sections from ischemic mice for the effects of pericytes on lumen diameter. Deconvolution microscopy revealed that α-SMA immunoreactivity colocalized with the pericyte markers NG2 and desmin, and the merged images showed an encircled lumen structure (Figure 5A). The specificity of the pericyte markers was confirmed by immunohistochemistry for monoclonal antibodies NG2, desmin, or α-SMA (Additional file 1: Figure S1). 

By double immunostaining for laminin and α-SMA, we compared mice treated with SB-3CT to vehicle-treated animals in both the ischemic cortex and the contralateral region. This experiment examined the effects of SB-3CT on gelatinase-associated laminin degradation and changes in the lumen diameter by pericytes. Pericytes were seen as annular cells surrounding the vascular lumen in the non-ischemic cortex (Figure 5A), with average lumen diameters of 5 ~ 10 μm. We further observed significant decreases in laminin-positive pericytes (Figures 5B and 5C) and pericyte-encircled lumen diameters in the ischemic penumbral cortex compared to the contralateral regions (Figure 5D). A higher magnification of the α-SMA-positive pericytes showed pericyte lumen contraction after embolus-induced cerebral ischemia (Additional file 2: Figure S2), suggesting that embolic ischemia caused loss of α-SMA-positive pericytes and increased pericyte lumen contraction. In the ischemic penumbral cortex, SB-3CT treatment protected laminin-positive pericytes by significantly increasing their numbers and the average pericyte lumen diameter (Figures 5C- 5D). These results indicate that SB-3CT can reduce the loss of laminin-positive pericytes, relieve them from ischemic contraction and restore microvascular patency, thus protecting against neurovascular impairment in embolus-induced focal ischemia in mice.

### SB-3CT attenuates gelatinase-mediated impairment of the neurovascular unit and reduces intracerebral hemorrhage in ischemic mice

**Figure 6:**
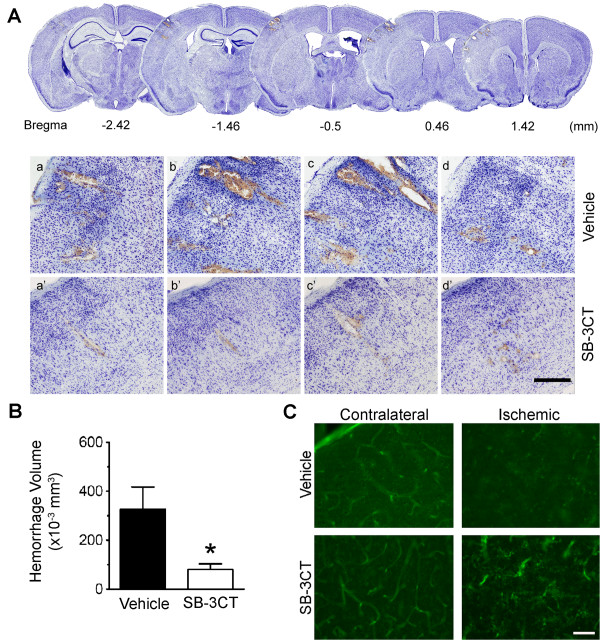
**SB-3CT attenuates gelatinase-associated loss of endothethial cells and decreases ICH. a** Representative brain sections stained with cresyl violet reveal secondary micro-hemorrhages in the ischemic cortex after embolic stroke in mice. Top panels: A lower magnification of serial sections, which are separated in 960 μm intervals for adjacent sections. Lower panels: Higher magnification of the cresyl violet-stained brain sections (separated in 200 μm intervals for two adjacent sections), in the ischemic cortex 1 day after treatment with either vehicle (a-d) or SB-3CT (a’-d’). Scale bar, 300 μm. **b** Quantification of hemorrhagic volume. Micro-hemorrhages were evaluated in cresyl violet-stained brain sections by bright-field microscopy. ICH volumes were quantified using the stereology technique, which utilizes systematic sampling of 25–30 serial sections per brain, each section separated by 200-mm along the anteroposterior axis of the mouse brain. Analysis of the ICH volumes revealed that SB-3CT significantly reduced ICH volume in ischemic brains (n = 3), *p* < 0.05 by one-tailed Student’s *t*-test, and data are expressed as means ± SEM. **c** Fluorescence staining shows loss of lectin-positive endothelial cells in the embolic ischemic cortex. SB-3CT blocks deformation of endothelial cells in the ischemic cortex. Scale bar, 50 μm.

We and others have shown that activated MMP-9 directly induces neuronal apoptosis and hemorrhage, and that SB-3CT treatment can protect against neurotoxicity, BBB disruption and hemorrhage [29,31,38]. In this study, we evaluated whether SB-3CT had any effect on reducing secondary hemorrhages after embolic ischemia in mice by examining embolic stroke-induced hemorrhages using brain sections stained with cresyl violet, as described previously [39]. Cresyl violet-stained brain sections revealed scattered secondary micro-hemorrhages in the ischemic cortex (Figure 6A). We further quantified micro-hemorrhagic volumes with the stereology technique [39]. Results show that while the average micro-hemorrhage in the ischemic brains was more than 300 μm^3^, SB-3CT treatment significantly decreased ICH volumes to less than 100 μm^3^ (Figure 6B). 

Next, we investigated the effects of SB-3CT on the integrity of neurovasculature by examining microvascular endothelial cells using the specific endothelial marker glycoprotein *Lycopersicon esculentum* (tomato) lectin-FITC and the BBB tight-junction protein ZO-1. Fluorescence staining revealed that microvascular lectin-FITC immunoreactivity colocalized with the well-documented antibody marker CD31 for endothelial cells (Additional file 3: Figure S3). We observed loss of microvascular endothelial cells in the embolus-induced ischemic cortex (Figure 6C). In contrast, SB-3CT protected against endothelial deformation and antagonized the loss of lectin-positive brain capillary endothelial cells. We also observed gelatinase-mediated degradation of the tight-junction protein ZO-1 and fragmentation of the ECM laminin in the ischemic cortex (Additional file 4: Figure S4). These findings were similar to those previously reported by us and others [13,15,17,29,38]. Taken together, these data indicate that an impaired neurovasculature leads to secondary micro-hemorrhages and that SB-3CT protects against gelatinase-mediated neurovasculature impairment in embolic ischemia.

### Repeated-dose SB-3CT treatment attenuates degradation of neuronal laminin, protects neurons from ischemia and improves neurobehavioral outcomes after embolic MCA occlusion

**Figure 7:**
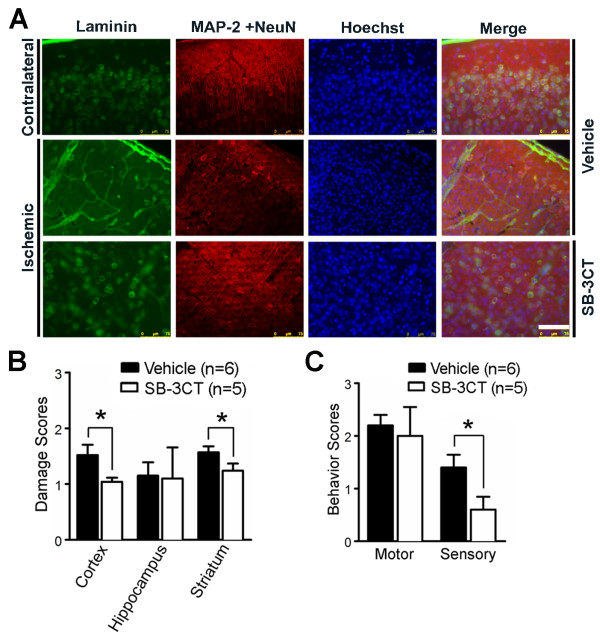
**SB-3CT treatment for 7 days attenuates degradation of neuronal laminin, protects neurons from ischemic cell death, and ameliorates neurobehavioral outcomes after embolic MCA occlusion.** Mice were ip injected with SB-3CT or vehicle 2 and 4 hours after MCA occlusion, and then once a day from post-ischemia days 1 to 6. **a** Immunohistochemistry of representative cortical regions for the ECM component laminin (green) and neuronal markers, MAP-2 and NeuN (red), with DNA counterstain Hoechst 33324 (blue). Scale bar, 75 μm. Representative images revealed that there was loss of laminin-positive neurons after embolus-induced cerebral ischemia, while SB-3CT significantly attenuated neuronal laminin degradation and protected neurons from ischemic cell death. **b** Quantification of neuronal damage based on cresyl violet staining. Cellular damage in each region was scored as 0, normal (no apparent neuronal cell death); 1, fewer than 1/3 cells damaged; 2, more than 1/3 but less than 2/3 damaged; and 3, more than 2/3 of cells dead. Damage was determined 7 days after MCA occlusion. **c** Repeated-dose administration of SB-3CT improves sensory functions. *, *p* < 0.05 by one-tailed Student’s *t*-test for comparisons of the vehicle- vs. SB-3CT-treated groups. Number of animals (n) in each group is indicated in parenthesis, and data are expressed as means ± SEM.

Because of the unique mechanism of action of SB-3CT and its selectivity for gelatinases, repeated-dose SB-3CT treatment for seven days significantly antagonized neurovascular impairment by attenuating gelatinase-mediated neuronal laminin degradation (Figure 7A), and protecting neurons from cell death, as observed by cresyl violet staining (Figure 7B) and Fluoro-Jade B for neurodegeneration (Additional file 5: Figure S5). SB-3CT also ameliorated the mNSS 14-point neurobehavioral outcomes, particularly the sensory functions in the embolus-induced ischemia brains (Figure 7C).

## Discussion

In the present study, we investigated the selective gelatinase inhibitor SB-3CT in a mouse model of embolus-induced “permanent” focal cerebral ischemia. We demonstrated the following key findings: (1) the autologous blood clot embolus model is a reliable, physiologically induced focal ischemia model, highly relevant to thromboembolic stroke in humans; (2) the selective gelatinase inhibitor SB-3CT can inhibit MMP-9 activity *in vivo*, attenuate ECM basement membrane laminin degradation, prevent pericyte lumen from contraction, and protect pericytes and endothelial cells, thus playing an important role in preserving neurovascular integrity and reducing hemorrhage; and (3) repeated-dose SB-3CT treatment can counteract degradation of neuronal laminin, protect neurons from ischemic cell death, and ameliorate neurobehavioral outcomes after embolic MCA occlusion in mice.

The rodent embolus model has distinct advantages in testing anti-thrombolytic and neuroprotectant agents for cerebral ischemia and is recommended by the Stroke Therapy Academic Industry Roundtable (STAIR) [40]. In our embolic model, the MCA supporting the brain region blocked by the embolus shows insufficient perfusion compared to the contralateral hemisphere. This model mimics the pathological conditions of an autologous clot-induced MCA occlusion. Initially, one of our goals was to test the reliability of the embolus model in comparison with the widely used filament model [29,34-36]. We also aimed to extend previous studies with the selective gelatinase inhibitor SB-3CT in the embolus model in mice, and further evaluate the suitability of the selective gelatinase inhibitor after two doses on day 1 or repeated-dose administration for 7 days. Results from this study indicate that the embolus model can be used to test thrombolytic or neuroprotective agents.

In the developing and adult brains, MMP proteolytic activity is known to play roles in modulating diverse physiological functions in the central nervous system, such as ECM remodeling, myelin turnover, axon outgrowth, angiogenesis, long-term potentiation, and synaptic plasticity in memory and spatial learning [5,7,25,26]. However, these enzymes also play important roles in mediating pathological processes, especially after stroke or traumatic injuries [5,7]. Elevated MMP-9 activity is correlated with BBB disruption, brain edema, hemorrhagic transformation, and neuronal apoptosis [2,4,12,13]. There is evidence indicating that specific inhibition of MMP activity during stroke onset or immediately following brain injury likely improves neurological outcomes. Lo and colleagues reported that minocycline, a broad-spectrum MMP inhibitor, could reduce neuronal cell death after ischemia and extend the thrombolytic time window [41]. Moreover, MMP-9 knockout can stabilize the BBB by preventing degradation of the tight-junction protein ZO-1 [17]. During ischemic stroke, MMP-9 inhibition also mitigates BBB disruption and reperfusion injury experimentally induced by the thrombolytic agent tPA, a therapeutic agent that can itself increase the risk of bleeding [6,14]. A recent clinical trial indicated that minocycline can lower plasma MMP-9 levels, even at 72 hours after stroke, and improve neurological outcomes in acute ischemic stroke patients treated with tPA [11]. In the present study, using the embolus-induced ischemia model, treatment with SB-3CT revealed an unexpected finding as compared to the control group. During ischemia, expression of inactive proMMP-9 increased significantly, a small portion of which was processed to active MMP-9, which manifests the pathological consequences of ischemia. When ischemic animals were treated with SB-3CT, we observed a general down regulation of proMMP-9 expression. This could be the consequence of a feedback process whereby active MMP-9 interacting with SB-3CT via its thiolate acts on an unknown downstream target to modulate the MMP-9 expression by transcription factors such as NF-kappa B and AP-1 [5].

MMPs have been implicated in beneficial roles in recovery following stroke. Shortly after ischemic insult, a cascade of events is initiated in attempt to repair the damage, a process similar to that found in wound healing [5,7]. Following injury, blood vessels are dependent on the plasminogen-activator system and on MMPs for their regeneration [42]. For example, Lo and colleagues showed that delayed inhibition of MMPs by broad-spectrum inhibitors is detrimental during the ischemic recovery stage seven days after cerebral ischemia, hindering brain repair in mice, and attenuating neurovascular regeneration in the penumbra [21]. Perhaps, a suitable balanced level of MMP-9 activity is important for vascular remodeling after spinal cord injury [22]. Therefore, extended inhibition of MMPs, especially through the use of broad-spectrum inhibitors, might prove deleterious [2,5].

In the present study, we observed that the mNSS scores of both motor and sensory functions after two-doses of SB-3CT on day 1 are significantly lower, compared to the vehicle-treated group (Figure 3C). Moreover, repeated-dose administration of SB-3CT for seven days appears to offer beneficial effects after embolic ischemic injury, as demonstrated by its ability to attenuate degradation of neuronal laminin, protect neurons from ischemia shown on cresyl violet stained brain sections and ameliorate neurobehavioral outcomes (Figure 7). These data are in line with the decrease in infarct volume and the ability of SB-3CT to protect the brain from ischemic damage. Measurement of the sensory functions showed significant improvement at the end of the seven-day SB-3CT treatment, but no further change in motor function (Fig. 7C). However, we observed that mice in the vehicle-treated group had significantly improved motor functions by seven days after ischemia, as indicated by a diminution in their motor function mNSS from a score of 5 at day 1 to a score of 2 at day 7. It has been shown that rodents are able to recover gradually in motor function after ischemic insult despite persistence of the infarct area [43,44]. These observations for the improvement in motor function might be due to compensatory effects of the endogenous neurogenic response and/or subsequent retrieval of interhemispheric functional connectivity within the sensorimotor system in rodents. Nevertheless, repeated-dose administration of SB-3CT for seven days offered beneficial effects, such as protecting neurons and ameliorating neurobehavioral functions in stroke outcomes relative to vehicle-treated mice. On the other hand, delayed treatment with the broad-spectrum MMP inhibitors FN-439 (day 3 or 7) and BB-94 (day 7) significantly worsened infarct volumes [21]. Since these broad-spectrum MMP inhibitors are peptidomimetics and they were administered intraventricularly [21], they are unlikely to cross the BBB. In contrast, SB-3CT crosses the BBB in healthy animals (unpublished data). These data suggest the benefit of selective gelatinase inhibitors over broad-spectrum MMP inhibitors [27].

Using the embolic model for “permanent” MCA occlusion in mice, we documented the protective effects of SB-3CT on the neurovascular unit. Since SB-3CT is a small molecule that distributes to the brain, it enters the brain via the unaffected vasculature, and diffuses to the infarct regions to manifest its inhibitory activity. In this study, we demonstrated the protective effects of SB-3CT despite the fact that the embolus remained intact in the brain during the course of treatment. As such, this inhibitor is considered a PAT agent. The basis of the selectivity of SB-3CT is the ability of gelatinases to catalyze a requisite thiirane-ring opening of the SB-3CT structure [45]. The thiolate, formed only within the active site of gelatinase, is capable of coordinating with the active-site zinc ion, and precipitates potent “slow-binding” inhibition, a hallmark of this class of inhibitors. Inhibition inherent to this class of compounds suppresses pathological activity of the target gelatinase, which is activated during the diseased state [27]. Under this condition, the inhibitory effect of SB-3CT is limited to gelatinases. In addition to stroke, this mechanism-based, selective gelatinase inhibitor can exhibit promising efficacy on other diseases. For example, recent studies demonstrated that SB-3CT attenuates BBB damage by decreasing oxygen-glucose deprivation-induced occludin degradation in early ischemic stroke, protects against oxidative stress-mediated apoptosis after spinal cord injury, and increases proliferation of NG2 progenitor cells after spinal cord injury [30,32,46]. Another study showed that SB-3CT could prevent laminin degradation and ameliorate neuronal death in a rat model of subarachnoid hemorrhage [31]. Taken together, these studies along with the recent findings of SB-3CT on neurovasculature [8,20], indicate that SB-3CT has promise in the treatment of gelatinase-mediated diseases. The inhibitor also exhibits significant efficacy in maintaining brain neurovascular integrity and protective effects on neuroinflammation against degradation of ECM components, pericyte lumen contraction, BBB disruption, neuronal apoptosis, and hemorrhagic transformation in brain and spinal cord injuries.

Brain function and neuronal viability are dictated by the adequate delivery of oxygen and glucose through cerebral microvessels. Because of their small diameter and relatively low flow velocity, microvessels are prone to occlusion by thromboembolism. Substantial evidence indicates that pathological MMP-9 activity after cerebral ischemia degrades components of the ECM and tight-junctions, thus contributing to microvessel and BBB leakage [13,17]. We previously showed that MMP-9 activation is essential for degradation of laminin after focal cerebral ischemia [29]. Pericytes are known to play a role in modulating capillary diameter by constricting the vascular wall, a process that can obstruct capillary blood flow during ischemia in the adult brain [20]. In this study, we observed a significant decrease in laminin-positive pericytes, and an increase in pericyte lumen contraction in the ischemic cortex (Figure 5). Because oxidative and nitrosative stress might promote capillary constriction after ischemia, and *S*-nitrosylation contributes to MMP-9 activation after stroke [16], it is reasonable to consider pericytes as critical targets for MMP-9 proteolytic activity after cerebral ischemia. The ability of SB-3CT to protect laminin-positive pericytes, and inhibit lumen contraction after embolic MCA occlusion is critical for its efficacy in restoring microvascular patency and protecting against neurovascular impairment. Moreover, the ability of SB-3CT to attenuate gelatinase-mediated damage of endothelial cells and BBB tight-junction ZO-1 and to protect against endothelial deformation is important in its ability to decrease ICH in ischemic mice.

## Conclusions

In summary, we used an embolic cerebral ischemia mouse model to evaluate SB-3CT, a mechanism-based selective and potent gelatinase inhibitor. We demonstrated MMP-9 activation and neurovasculature impairment in stroke, and showed the ability of SB-3CT to inhibit MMP-9 activity *in vivo*, which resulted in maintenance of laminin, antagonism of pericyte contraction and loss, and preservation of laminin-positive pericytes and endothelial cells. Thus, SB-3CT rescued neurons from apoptosis and prevented ICH. Selective targeting of MMP-9 antagonizes disruption of the neurovascular unit and protects against brain damage and vessel constriction. Taken together, these results strongly indicate that thiirane class of gelatinase selective inhibitors holds great promise as therapeutic agents for the treatment of stroke.

## Methods

### Animal models of focal cerebral ischemia in mice

Adult male C57Bl/6 J mice (The Jackson Laboratory, Bar Harbor, Maine, USA), 6–9 weeks of age and weighing 20–28 g were housed in a 12 hours light/dark cycle and permitted food and water intake *ad libitum* . All groups in the animal experiments were performed in a randomized, blinded manner at the University of Missouri according to an institutionally approved protocol in accordance with the National Institutes of Health Guide for the Care and Use of Laboratory Animals. Mice were anesthetized with isoflurane (1–1.5%) masked with a gas mixture of 30% oxygen and 70% nitrogen. Rectal temperatures were maintained at 37 ± 0.5°C with animals placed on a thermostat-controlled heating pad.

Two mouse models of focal cerebral ischemia were used: an embolic model using autologous clots to occlude the MCA and a filament model described previously [29,34-36]. For the latter, C57BL/6 J mice were subjected to right MCA occlusion by insertion of a 6–0 monofilament to block the MCA for 2 hours, and followed by filament withdrawal for arterial reperfusion. For the embolic model, homologous blood clots were prepared by a modification of earlier methods [34]. Briefly, femoral arterial blood from a donor mouse was withdrawn into a 3-foot long PE-50 polyethylene tubing, incubated at 37°C for 2 hours, and cooled at 4°C overnight to allow the formation of fibrin-rich blood clots. Blood clots were then washed with sterile phosphate buffered saline (PBS) in a 2-foot long PE-10 tubing for 6 ~ 8 rounds. After ejection from the PE-10 tubing, clots were cut into 10-mm long (0.02 μl) pieces. A single piece autologous clot was inserted via a PE-10 catheter with a modified tip of ~0.20 mm outer diameter from the external cerebral artery into the internal cerebral artery lumen to occlude the MCA. The catheter was immediately removed after injection. Both models required approximately 15–20 min (no longer than 20 min) from the start of anesthesia to completion of the surgery; animals were not included in the subsequent study if the surgery took over 20 min. A total of 94 mice were used in the subsequent study.

### SB-3CT administration

SB-3CT, a discovery from the Mobashery laboratory, was synthesized for this study by reported methodology [47]. Mice were divided into four groups: vehicle-treated group and SB-3CT-treated one with treatment for either one day or seven days after embolic MCA occlusion. SB-3CT (12.5 mg/mL) was freshly dissolved in 25% DMSO/65% PEG-200/10% water and filtered through an Acrodisc syringe filter with a 0.2 μm, 13-mm diameter sterile hydrophobic PTFE membrane (VWR, West Chester, PA). Mice were ip injected with 2 μL/gram body weight of this solution (equivalent to 25 mg/kg) 2 hours after embolic ischemia, followed by an additional dose at 4 hours. In repeated-dose treatment conditions, the same dose of SB-3CT was ip administered 2 and 4 hours after embolic ischemia, followed by once daily from post-ischemia day 1 to 6. Earlier work indicated that ip administration of SB-3CT does not alter mean arterial blood pressure, pH, PCO_2_, and PO_2_[29].

### Monitoring of regional cerebral blood flow by laser doppler flowmetry

A laser Doppler probe (Moor Instruments, Devon, UK) was used to continuously monitor rCBF to ensure MCA occlusion. rCBF was expressed as a percentage of pre-ischemic baseline values. A total of 86 mice were used for embolic ischemia and approximately 80% of the mice showing sustained MCA blockage of less than 25% of pre-ischemic rCBF baselines were included in the subsequent study.

### Neurobehavioral assessment

Analysis of neurobehavioral deficits was conducted by individuals blinded to coded treatment groups. Animals underwent neurological evaluation using the 14-point mNSS on days 1 and 7 after embolic MCA occlusion. Neurobehavioral outcomes of the mNSS include a composite of motor, sensory and reflex tests, as described [37]. In scoring injury, one score point was awarded for the inability to perform a test or for the lack of a tested reflex; thus, the higher the score, the more severe the injury. Neurological function was graded on a scale of 0–14 (0 = normal, 14 = maximal deficit).

### Measurement of infarct volume

After neurological assessment, mice were sacrificed with an overdose of isoflurane and transcardially perfused with PBS to remove intravascular blood and brains were rapidly harvested. Coronal brain sections (1 mm thick) were stained with 2,3,5-triphenyltetrazolium chloride (TTC, Sigma, St. Louis, MO). Infarct volumes were quantified using the ImageJ analysis software, where TTC lesion areas from each slice were integrated to yield total ischemic lesion volumes. To minimize errors associated with edema during tissue processing for histological analysis, infarct volume is presented as a percentage of infarct volume relative to the volume measured in the contralateral hemisphere (indirect volume calculation) [21,29,35].

### Zymographic assays of gelatinolytic activity

Gelatin zymography was carried out as described [16]. Briefly, gelatinases were extracted in Tris-buffered saline, pH 7.6, followed by affinity precipitation with gelatin-Sepharose 4B. Bound material was released from the beads using 10% DMSO; the samples were analyzed by electrophoresis in a 10% gelatin zymogram gel under non-reductive conditions, followed by incubation overnight at 37°C in Tris buffer, pH 8, with 5 mM CaCl_2_. As a control, EDTA was added to other samples to inhibit all MMP activities. Gels were stained with Coomassie blue and digitized for densitometry analysis.

### Histological staining and assessment of brain damage

At days 1 or 7 after embolic ischemia, mice were sacrificed and brains were processed for histochemical staining using the stereology technique as described [36,39]. Briefly, brains were dissected and submerged in 4% paraformaldehyde overnight at 4°C. Coronal sections were serially cut through the entire brain at 40 μm intervals with a vibrotome (VT1200S, Leica Microsystems, Inc., Bannockbum, IL), and a total of 120–150 tissue sections were sequentially collected into a 24-well plate (e.g., a distance of 960μm in two adjacent sections in each well). every 5th section was reserved for histochemical staining with cresyl violet to analyze neuronal cell death and score brain damage. Stained sections were examined using an Olympus BX-41 upright pathology microscope (Olympus America Inc., Center Valley, PA). The grading scale for neuronal damage in each region was scored as previously described [36]: 0, normal (no apparent neuronal cell death); 1, fewer than 1/3 cells damaged; 2, more than 1/3 but less than 2/3 damaged; and 3, more than 2/3 of cells dead.

### Quantification of hemorrhagic volume

Micro-hemorrhages were quantified in cresyl violet-stained serial sections with stereology analysis technique [39], which utilizes systematic sampling of 25 to 30 serial sections per brain, each section separated by 200 μm along the anteroposterior axis of each brain. Spontaneous ICH after embolic ischemia was examined as described [15], using a 4x objective lens. Additional validation was carried out by capturing images using a 20x objective and micro-hemorrhage area was measured using the ImageJ analysis software. Care was taken to examine each serial section to include multiple hemorrhage areas per section. Hemorrhage area for each animal was summed and then hemorrhage volumes were calculated by integrating the appropriate area with the section interval thickness (200 μm) and expressed as μm^3^.

### Fluorescence labeling and immunohistochemistry

Cerebral vascular perfusion patterns were examined after embolic cerebral ischemia using high-molecular weight fluorescein-dextran. A modified PE-10 catheter connected to a 1-cc syringe was inserted into the right femoral vein and a solution of FITC-dextran [0.1 mL of 50 mg/mL in PBS; molecular weight (MW) 2,000 KDa, Sigma Chemical Co, St. Louis, MO] was administered for 30 minutes before the animal was sacrificed. Mice were decapitated and the brains were rapidly removed and placed in 4% paraformaldehyde for 24 hours. Coronal sections (100 μm thickness) were cut with a vibrotome and post-fixed in 4% paraformaldehyde. For immunohistochemistry, 40 μm thick brain sections were labeled for microvessel endothelial cells using the specific glycoprotein *Lycopersicon esculentum* (tomato) lectin conjugated to fluorescein (1:500, FL-1171; Vector Labs, Burlingame, CA) or immunostained with the following antibodies: the endothelial marker rat anti-CD31 (1:50, 550274; BD Biosciences, San Diego, CA); the neuronal markers MAP-2 (1:200, M4403, Clone HM-2; Sigma Chemical Co, St. Louis, MO) and NeuN (1:200, MAB377, Clone A60; Millipore-Chemicon, Temecula, CA), and Fluoro-Jade B (0.0012%, AG310; EMD-Millipore, Temecula, CA) fluorescence staining for degenerating neurons; the ECM basement membrane component laminin (1:200, L9393; Sigma Chemical Co, St. Louis, MO); and the pericyte markers―NG2 (1:200) described [46], desmin (1:50, M0760, Clone D33; DakoCytomation, Carpinteria, CA), and α-SMA (1:400, MS-113-P1ABX, Clone 1A4; Thermo Scientific-Lab Vision, Fremont, CA); and mouse anti-ZO-1 antibody recognizing BBB tight-junction (1:50, 33–9100; Invitrogen, San Diego, CA). Sections were then visualized with fluorophore-conjugated secondary antibodies (1:200, goat anti-mouse IgG-Alexa488, A11001; goat anti-mouse IgG-Alexa594, A11005; and goat anti-rabbit IgG-Alexa488, A110034; Invitrogen, San Diego, CA).

For double immunostaining, we incubated sections with Pro-Block (PBK125; ScyTek, Logan, UT) for 5 minutes to eliminate the need to match species with the fluorescence-conjugated antibody, followed by incubation with normal goat serum for 2 hours to block nonspecific binding before applying the primary antibody. Specificity controls included omitting the primary or secondary antibody. Sections were counterstained with nuclear DNA dye Hoechst 33342 [29]. Fluorescence images were analyzed with a Leica DMI 6000B fully automated epifluorescence microscope including AF6000 applications for deconvolution (Leica Microsystems Inc., Buffalo Grove, IL) or an Olympus XI-81 spin-disc scanning confocal microscope (Olympus America Inc., Center Valley, PA), and mosaic images were stitched using Montage analysis tools (Slidebook™ from 3I, Denver, CO) for image re-construction.

### Measurement of pericytes

We quantified α-SMA-positive pericytes in 4–5 randomized areas in the cortical penumbral region of the lesioned hemisphere and the corresponding areas of the contralateral side by counting approximately 1500 total cells in each hemisphere. We calculated percentages of α-SMA-positive pericytes and both α-SMA/laminin-positive pericytes relative to total cells visualized by the Hoechst dye staining [8,19,20] and measured the average lumen diameters of the long- and short-axis of α-SMA labeled pericytes using the ImageJ software. The long diameter (ΦL) is defined as the longest diameter in the lumen circles, while the short diameter (ΦS) is defined as the perpendicular bisector of the long diameter. We compared percentages of laminin-positive pericytes relative to total cells visualized by staining the nuclear DNA of cells with Hoechst dye, and the overall (laminin-positive and -negative) pericyte-encircled lumen diameters.

### Statistics

All experiments were performed in a randomized blinded manner, with data analysis and experiments performed by separate investigators. Each n number represents an individual animal for each experiment. Data were analyzed using Prism 5 software (GraphPad Software, La Jolla, CA) by either paired or unpaired one-tailed Student's *t*-test for two-group comparisons. All data are presented as means ± SEM. Statistical significance was set at *p* < 0.05, 0.01 or 0.001, respectively.

## Competing interests

The authors declare that they have no competing interests.

## Authors' contributions

Author contributions: JC and ZG conceived and designed the project; ML, MC and SM prepared the test compound; JC, SC, CZ, FM, WW, RH, OH, TL, GJB, and ZG performed the experiments; JC, SC, CZ, FM and ZG analyzed data, JC, SC, and ZG wrote the manuscript with significant input from MC, SM and GYS. All authors have read and approved the final manuscript.

## Supplementary Material

Additional file 1 **Figure S1.** Testing specificity of the pericyte markers α-SMA, NG2 and desmin by Immunohistochemistry. NG2 or desmin monoclonal antibody (mAb) was used to detect pericytes with anti-mouse IgG-Alexa488 (GαM Alexa-488; green), followed by immunostaining with α-SMA mAb and visualized with anti-mouse IgG-Alexa594 (GαM Alexa-594; red). Various immunohistochemistry conditions for the double immunostainings with dual mAbs were examined by omitting either the primary mAbs with the nuclear DNA dye Hoechst for counter-staining the total cells. Immunohistochemistry analysis reveals colocalization of α-SMA-positive cells with cells expressing the pericyte markers NG2 (A) and desmin (B). Scale bars: 50 μm. Click here for file

Additional file 2 **Figure S2.** Effect of SB-3CT on α-SMA-positive pericytes after embolic ischemia. Pericytes were immunstained with α-SMA mAb. Comparing images of α-SMA-positive pericytes in the ischemic cortex with those in the corresponding contralateral region reveals that treatment with SB-3CT partially protects pericytes from ischemia-induced cell loss and lumen contraction after embolic MCA occlusion in mice. Scale bars: 20 μm. Click here for file

Additional file 3 **Figure S3.** Colocalization of dual endothelial markers. Immunostaining of endothelial marker lectin (in green) with the endothelial marker CD31 (in red) reveals colocalization of lectin-positive cells with the endothelial marker CD31. DNA counterstaining with Hoechst 33324 reveals total cells. Scale bars: 50 μm. Click here for file

Additional file 4 **Figure S4.** Degradation of the tight-junction protein ZO-1 after embolic ischemia. Merged images with DNA counterstain by Hoechst 33324 reveals colocalization of laminin-positive microvessels (in green) with the BBB tight-junction marker ZO-1 (in red). Representative double immunostaining images in ischemic cortex penumbras are compared with the corresponding contralateral regions. There are degradation of the tight-junction protein ZO-1 and fragmentation of ECM laminin-positive microvessels in the ischemic cortex. Scale bars: 50 μm. Click here for file

Additional file 5 **Figure S5.** Protection against neurodegeneration by prolonged treatment with SB-3CT for 7 days in mice after embolic ischemia. Immunohischemistry using Fluoro-Jade B (in green) reveals neurodegeneration in the ischemic cortex 7 days after embolus-induced MCA occlusion in mice. SB-3CT treatment for 7 days significantly protects against cortical neurodegeneration. Scale bars: 50 μm. Click here for file
