# Supplementary figures and images for: Inhibition of MMP-9 by a selective gelatinase inhibitor protects neurovasculature from embolic focal cerebral ischemia
Source: Mol Neurodegener. 2012 May 15;7:21. doi: 10.1186/1750-1326-7-21 (PMC3500265; doi:10.1186/1750-1326-7-21)

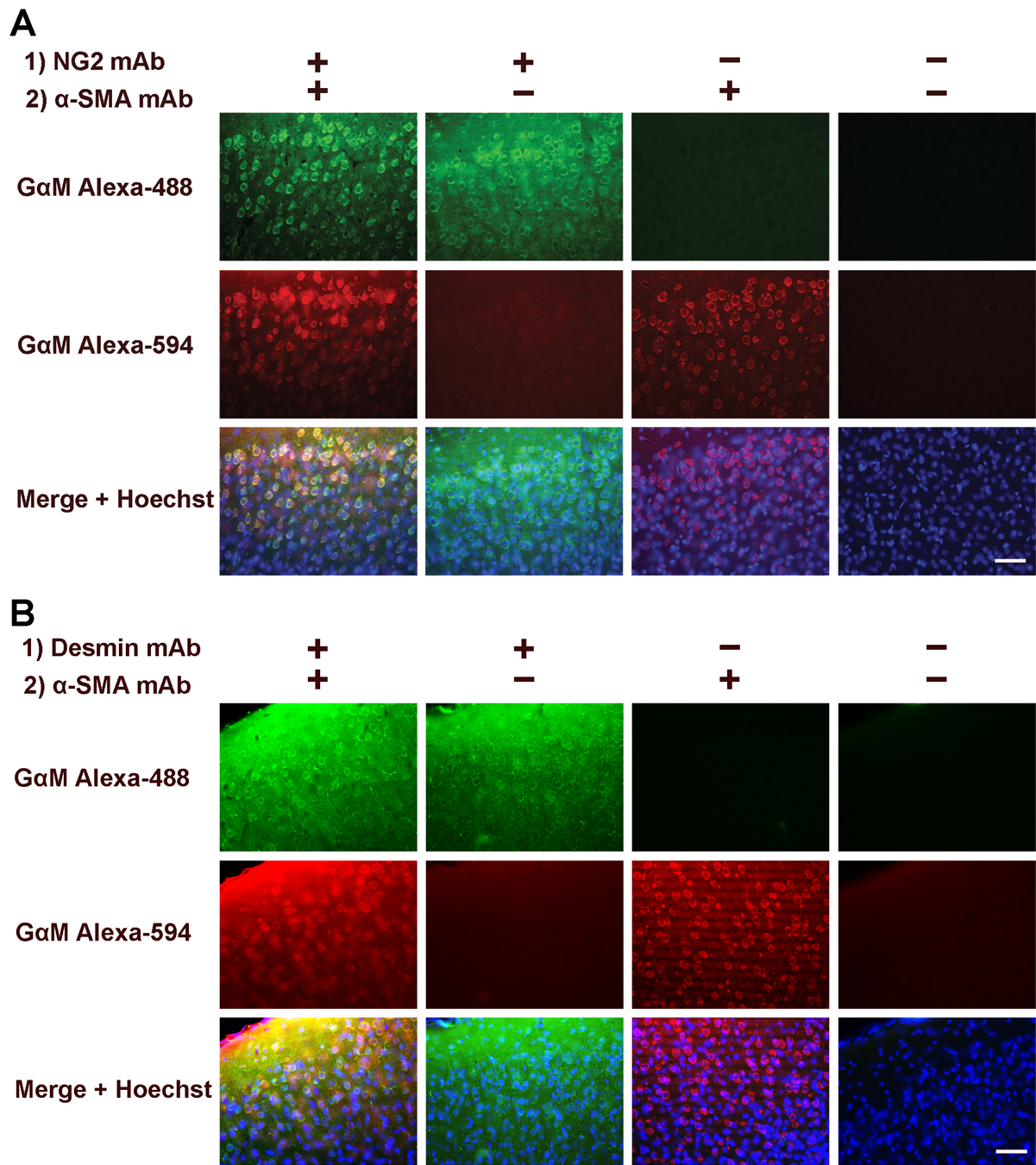

Supplement: Additional file 1 — Figure S1. Testing specificity of the pericyte markers α-SMA, NG2 and desmin by Immunohistochemistry. NG2 or desmin monoclonal antibody (mAb) was used to detect pericytes with anti-mouse IgG-Alexa488 (GαM Alexa-488; green), followed by immunostaining with α-SMA mAb and visualized with anti-mouse IgG-Alexa594 (GαM Alexa-594; red). Various immunohistochemistry conditions for the double immunostainings with dual mAbs were examined by omitting either the primary mAbs with the nuclear DNA dye Hoechst for counter-staining the total cells. Immunohistochemistry analysis reveals colocalization of α-SMA-positive cells with cells expressing the pericyte markers NG2 (A) and desmin (B). Scale bars: 50 μm. [file 1750-1326-7-21-S1.tiff]

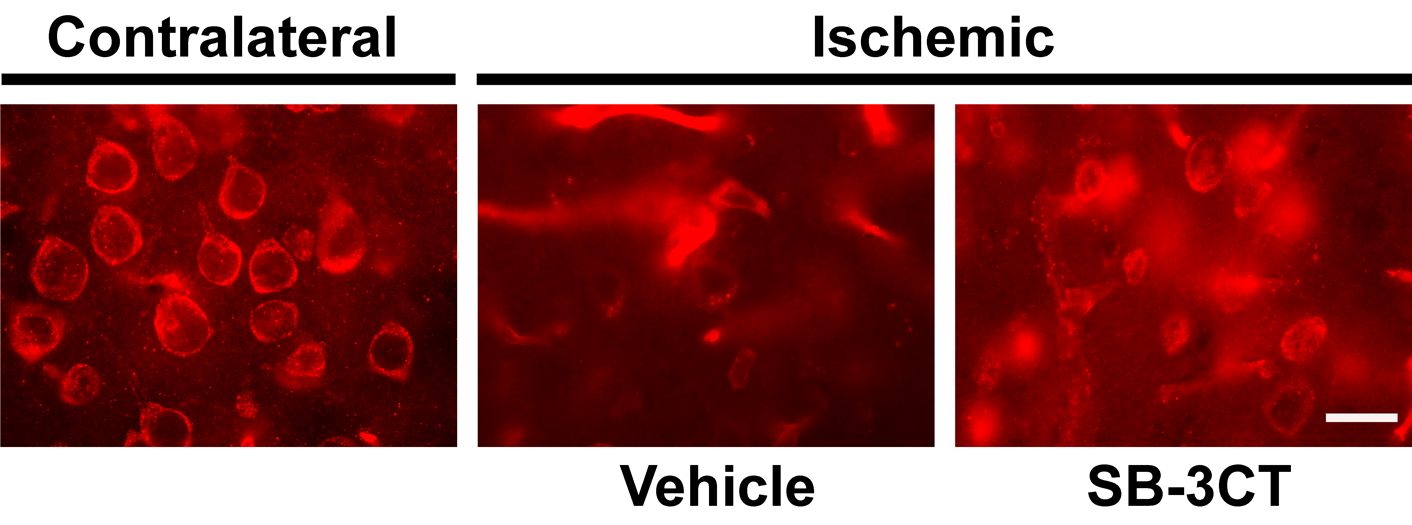

Supplement: Additional file 2 — Figure S2. Effect of SB-3CT on α-SMA-positive pericytes after embolic ischemia. Pericytes were immunstained with α-SMA mAb. Comparing images of α-SMA-positive pericytes in the ischemic cortex with those in the corresponding contralateral region reveals that treatment with SB-3CT partially protects pericytes from ischemia-induced cell loss and lumen contraction after embolic MCA occlusion in mice. Scale bars: 20 μm. [file 1750-1326-7-21-S2.tiff]

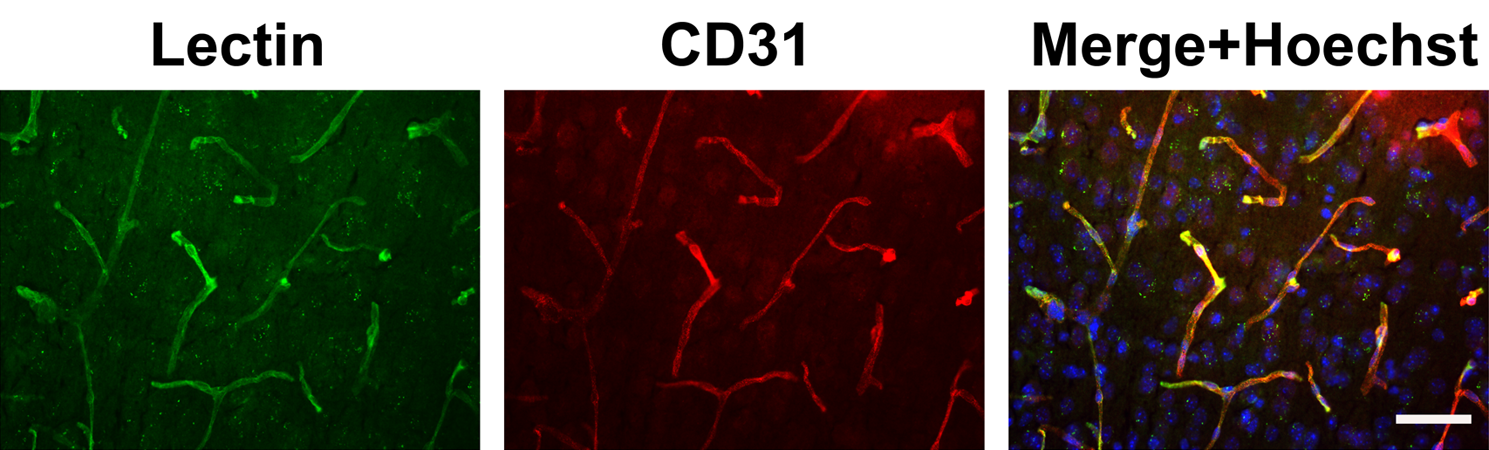

Supplement: Additional file 3 — Figure S3. Colocalization of dual endothelial markers. Immunostaining of endothelial marker lectin (in green) with the endothelial marker CD31 (in red) reveals colocalization of lectin-positive cells with the endothelial marker CD31. DNA counterstaining with Hoechst 33324 reveals total cells. Scale bars: 50 μm. [file 1750-1326-7-21-S3.tiff]

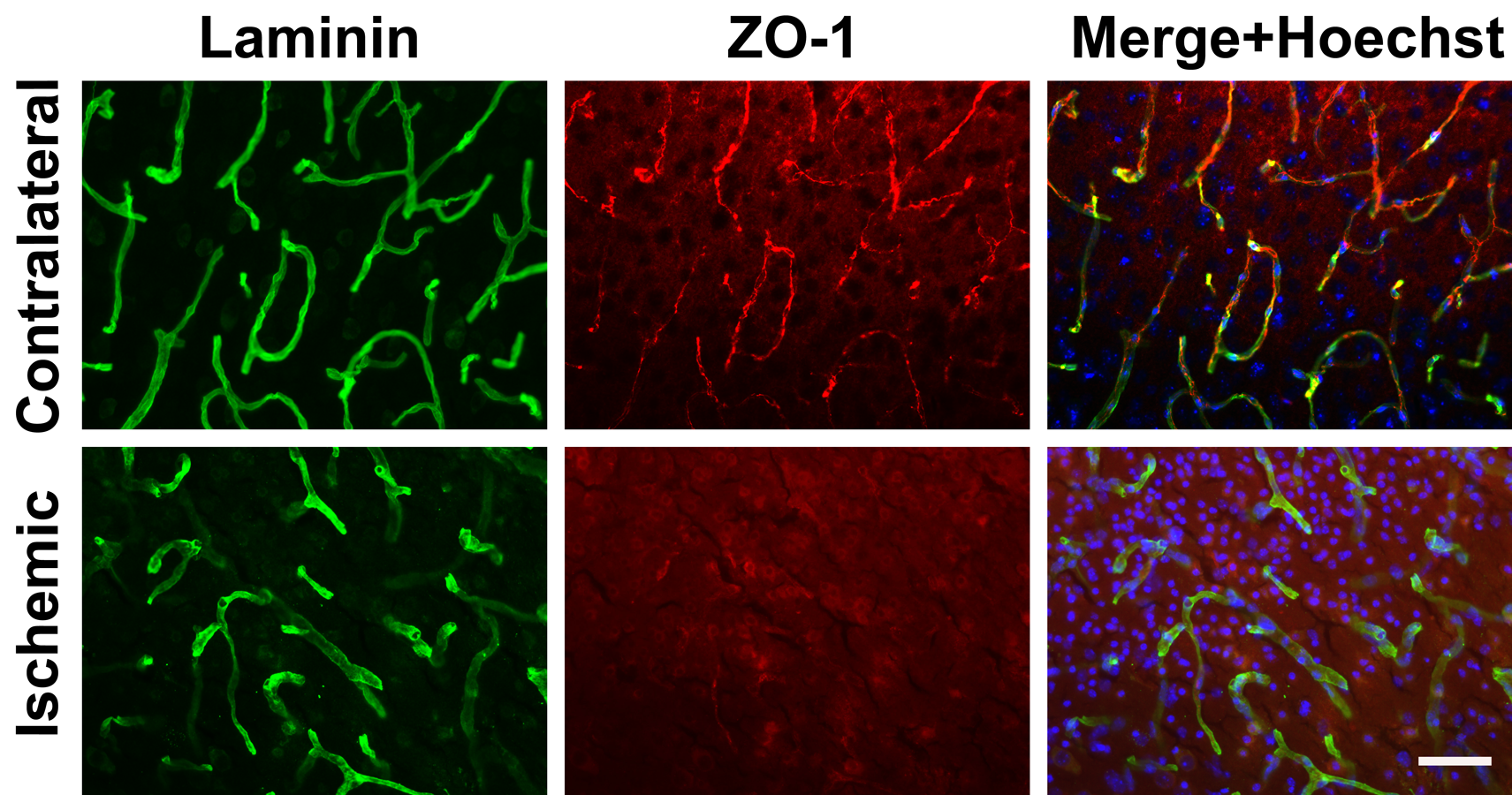

Supplement: Additional file 4 — Figure S4. Degradation of the tight-junction protein ZO-1 after embolic ischemia. Merged images with DNA counterstain by Hoechst 33324 reveals colocalization of laminin-positive microvessels (in green) with the BBB tight-junction marker ZO-1 (in red). Representative double immunostaining images in ischemic cortex penumbras are compared with the corresponding contralateral regions. There are degradation of the tight-junction protein ZO-1 and fragmentation of ECM laminin-positive microvessels in the ischemic cortex. Scale bars: 50 μm. [file 1750-1326-7-21-S4.tiff]

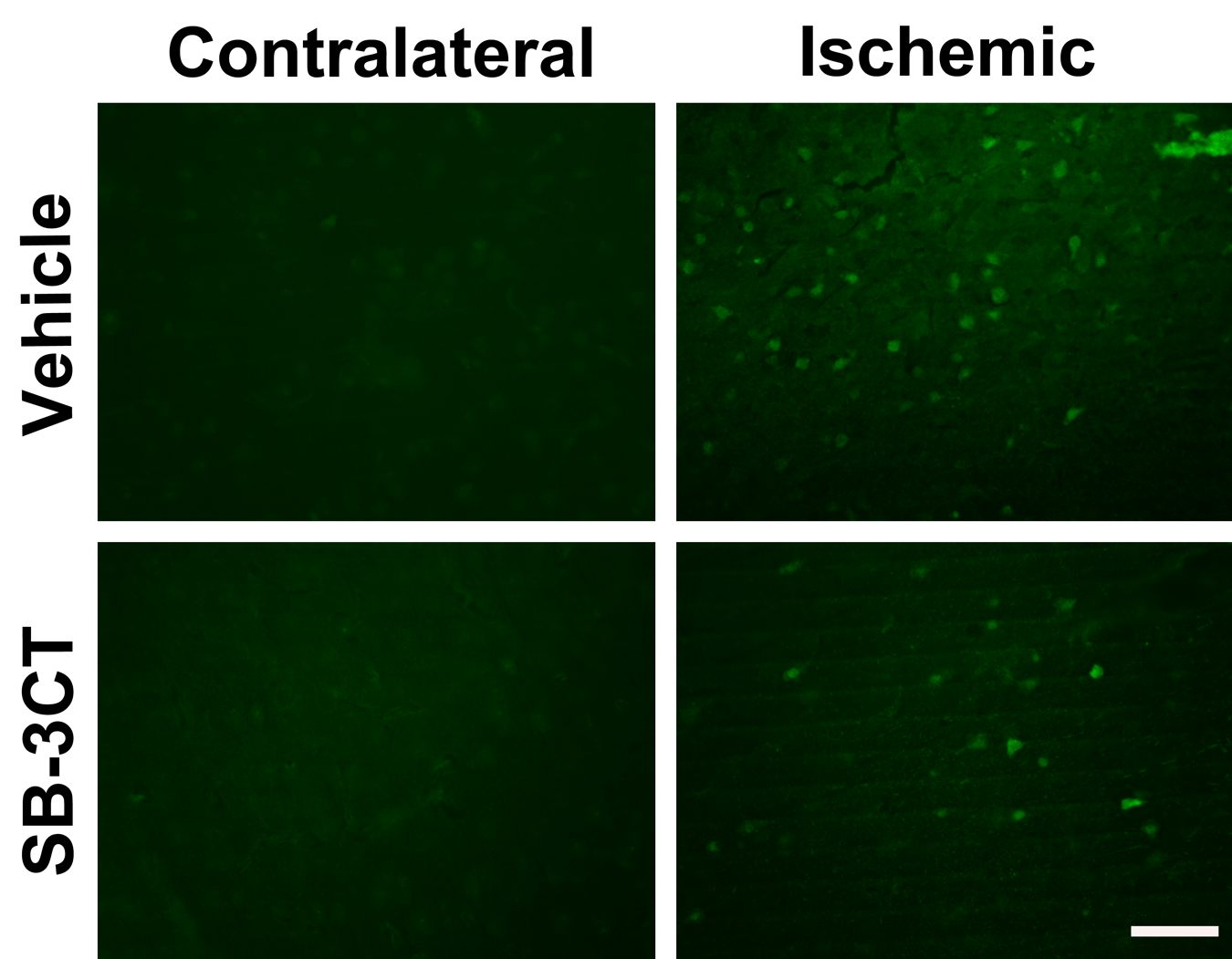

Supplement: Additional file 5 — Figure S5. Protection against neurodegeneration by prolonged treatment with SB-3CT for 7 days in mice after embolic ischemia. Immunohischemistry using Fluoro-Jade B (in green) reveals neurodegeneration in the ischemic cortex 7 days after embolus-induced MCA occlusion in mice. SB-3CT treatment for 7 days significantly protects against cortical neurodegeneration. Scale bars: 50 μm. [file 1750-1326-7-21-S5.tiff]
